# Supplementary material for: Linking wet-lab and genomic approaches for reliable detection of carbapenemase-producing Klebsiella pneumoniae in wastewater
Source: Front Microbiol. 2026 Jun 19;17:1821458. doi: 10.3389/fmicb.2026.1821458 (PMC13328271; doi:10.3389/fmicb.2026.1821458)
Supplement: Supplementary file 1 [file Table_1.DOCX]

**Supplementary Figures**

Werner et al. (2026)


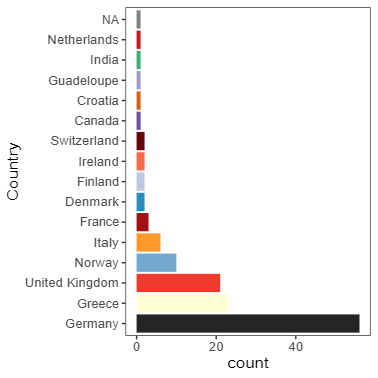


***Figure S1****.* ***Contributing countries to* Klebsiella pneumoniae *samples on Pathogenwatch.*** *Number of publicly available Pathogenwatch samples with a cgMLST allelic distance of fewer than 15 alleles to at least one of the 58 wastewater isolates from this study, grouped by country of origin (n= 133). Colours indicate countries.*


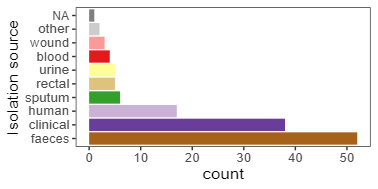


***Figure S2.*** ***Isolation sources of* Klebsiella pneumoniae *samples on Pathogenwatch.*** *Number of publicly available Pathogenwatch samples with a cgMLST allelic distance of fewer than 15 alleles to at least one of the 58 wastewater isolates from this study, grouped by isolation source (n= 133). Colours indicate isolation sources.*


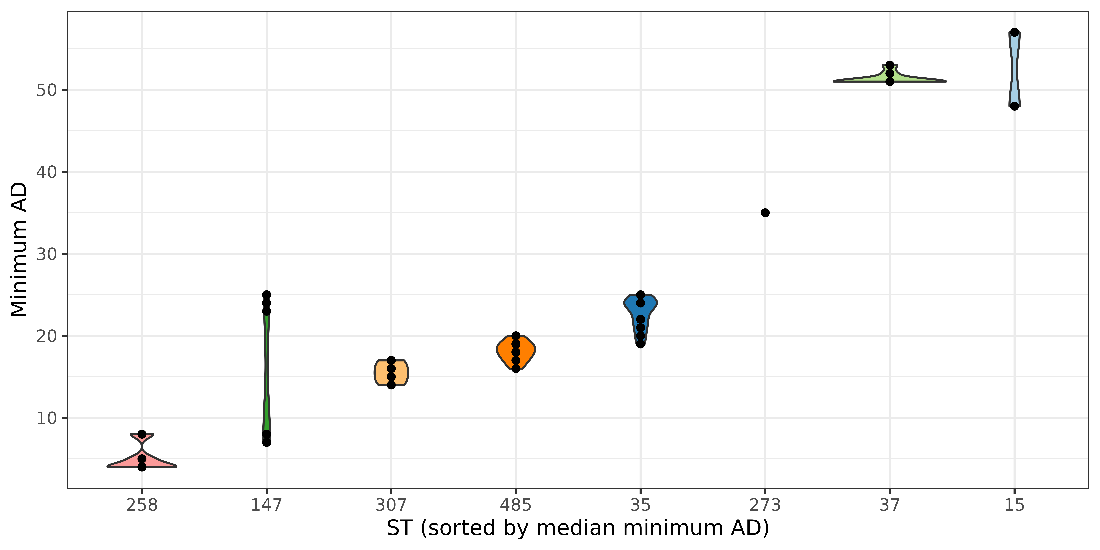


***Figure S3****.* ***Distribution of minimal cgMLST allelic distances between wastewater isolates and Pathogenwatch samples.*** *Violin plots showing the distribution of minimal cgMLST allelic distances (AD) between wastewater isolates from this study and publicly available Pathogenwatch samples, grouped by sequence type (ST). Each dot represents the minimal AD for a given wastewater sample.*


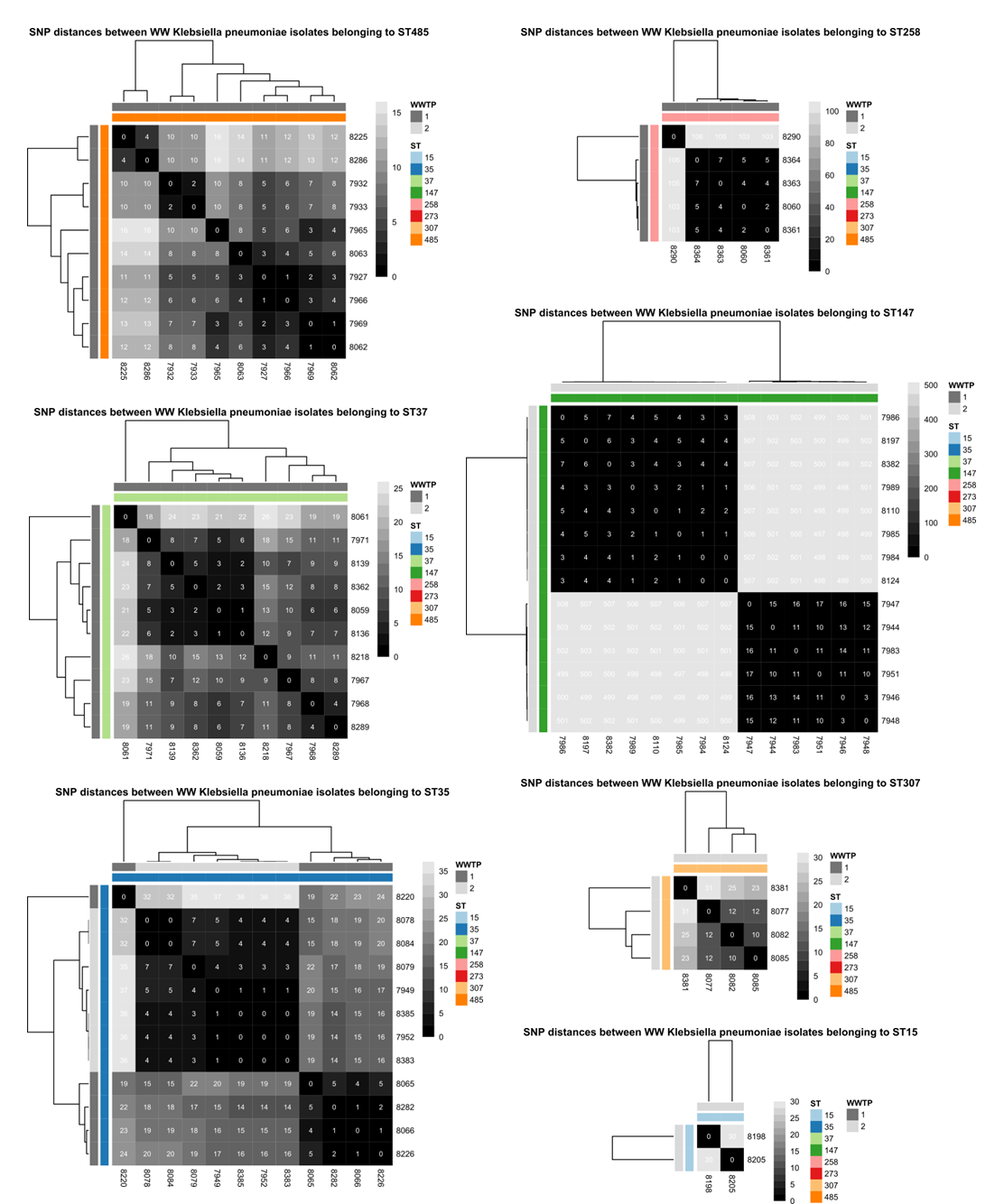


***Figure S4****.* ***SNP distances of WGS data of* Klebsiella pneumoniae *isolated from two WWTPs.*** *Heatmap of pairwise single nucleotide polymorphism (SNP) distances among* Klebsiella pneumoniae *wastewater isolates, based on snippy analysis. Reference genomes were samples 8286 (ST485), 8289 (ST37), 8383 (ST35), 8290 (ST258), 7985 (ST147), 8082 (ST307), and 8198 (ST15).*
